# Occupational incidence of bladder cancer amongst veteran ammunition technicians of the British Army

**DOI:** 10.1111/bju.16678

**Published:** 2025-03-22

**Authors:** Gareth Collett, Tim Bashford, Nik J. Whitehead, Ewa Kazimierska, Gokul Vignesh Kanda Swamy, Robert J. Jones, Mieke Van Hemelrijck, Richard T. Bryan

**Affiliations:** ^1^ Faculty of Ordnance, Munitions and Explosives University of Wales Trinity Saint David (UWTSD) Swansea UK; ^2^ Swansea Bay University Health Board Port Talbot UK; ^3^ School of Cancer Sciences, College of Medicine, Veterinary and Life Sciences University of Glasgow Glasgow UK; ^4^ Faculty of Life Sciences and Medicine, Transforming Cancer OUtcomes through Research (TOUR) King's College London London UK; ^5^ Bladder Cancer Research Centre, College of Medicine and Health University of Birmingham Birmingham UK

**Keywords:** bladder cancer, army, veteran, occupation, ammunition

**Abbreviations**
AT: ammunition technician
BC: bladder cancer
EOD: Explosive Ordnance Disposal
MIBC: muscle‐invasive bladder cancer
SIR: standardised incidence ratio

Bladder cancer is a common malignancy of the urinary tract [1], with occupational exposure the second most common modifiable risk factor after smoking [2]. Individuals involved in bomb disposal may be at risk of bladder cancer as explosive compounds such as nitro‐aromatics can be derivatives of benzene or amines [3, 4]. Such individuals include ammunition technicians (AT) within the British Army (the mainstay of high‐threat bomb disposal in the UK), where the risk of bladder cancer remains unknown and the association between exposure to explosives and bladder cancer is undetermined.

As a profession, AT personnel are exposed to potential carcinogens found in bulk explosives, their precursors, their detonation and degradation products, and the residue from the combustion of pyrotechnic compositions, ammunition, and associated packaging [5]. Pathways to exposure include inhalation, absorption, and ingestion. Hence, we sought to investigate the association between exposure to explosives and bladder cancer risk within the AT profession.

With ethical approval (University of Wales Trinity Saint David ethics reference EC1273 PG2), we used a questionnaire to survey the veteran AT network given security implications associated with the participation of serving individuals. The questionnaire (which can be made available upon request) was based upon up‐to‐date epidemiological evidence of risk factors [2] and sought to elucidate cancer occurrences. Veteran ATs with known contact details were approached on social media by the *Felix Fund Bomb Disposal Charity* between 1 March and 31 May 2024. The veteran cohort comprised 688 AT who had served within one particular Explosive Ordnance Disposal (EOD) squadron since 1970, representing 30% of the 2300 soldiers known to have trained and served to date (the largest possible sample size available). In this EOD squadron, AT personnel would have been exposed to all modalities of ammunition management prior to appointment, which includes manufacture, storage, inspection, maintenance, and disposal.

Given the vast array of explosives managed by the ATs, it was necessary to limit the scope of the survey. As such, nitro and nitramine explosives were highlighted as representative groups given that they are the most common explosives encountered within the global ordnance, munitions and explosives stockpile [6]—all serving and veteran ATs are exposed to them.

Responses to the survey were extracted and tabulated using descriptive statistics (Table 1). Standardised incidence ratios (SIRs) and 95% CIs for bladder cancer were then calculated by comparing the incidence in the AT cohort with the UK general population [7].

**Table 1:** Distribution of demographic and occupational risk factors by diagnosis of cancer within ATs.

Variable	Bladder cancer (*N* = 12)	Other cancer (*N* = 33)	No cancer (*N* = 158)
Age group (years), *n* (%)			
18–19	0	0	1 (<1)
20–29	1 (8)	0	1 (<1)
30–39	1 (8)	0	5 (3)
40–49	0 (0)	1 (3)	13 (8)
50–59	3 (25)	3 (9)	49 (31)
60–69	4 (33)	11 (33)	56 (35)
70–79	3 (25)	15 (45)	25 (16)
≥80	0 (0)	3 (9)	8 (5)
Risk factor			
Gender (descriptor), *n* (%)			
Male	12 (100)	33 (100)	157 (99)
Female	0 (0)	0 (0)	1 (<1)
White ethnicity (descriptor), *n* (%)	12 (100)	33 (100)	158 (100)
Smoker, *n* (%)	0 (0)	1 (3)	4 (2)
Ex‐smoker, *n* (%)	6 (50)	20 (61)	85 (54)
Non‐smoker, *n* (%)	6 (50)	12 (36)	69 (44)
Length of service, years, mean (SD)	23.45 (5.20)	21.06 (9.39)	20.83 (9.27)
Ammunition exposure (subjective ranking of highest to lowest exposure based on consultation with head of explosives profession), % (%)			
Exposure to nitro‐explosives during career (weekly exposure)	100 (67)	100 (24)	100 (16)
Disposal of OME during career (weekly exposure)	100 (42)	73 (21)	82 (28)
Storage of explosives during career (weekly exposure)	100 (67)	100 (48)	100 (54)
Inspection and safety management of explosives during career (weekly exposure)	100 (58)	100 (45)	100 (47)
Burning of OME during career (monthly exposure)	84 (33)	70 (27)	79 (23)
Manufacture of explosives during career (weekly exposure)	58 (18)	48 (6)	51 (10)

OME, ordnance, munitions and explosives.

We received 203 responses to the survey (30% of those 688 veterans approached). Of all cancers diagnosed within respondents, bladder cancer accounted for 27% (prostate, 22%; testicular, 4%; colon/bowel, 13%; throat, 4%; ureteric, 2%; myelodysplastic syndrome, 2%; and skin, 24%). In respondents with a bladder cancer diagnosis, a higher proportion reported weekly exposure to nitro‐aromatic explosives during their career (67%) than in other cancer (24%) and no cancer respondents (16%); a similar relationship was observed for weekly exposure during EOD tasks (42%, 21% and 28%, respectively).

Using the whole cohort of 688 to compare bladder cancer incidence with the UK general population, SIRs showed increased occurrence of bladder cancer amongst AT (SIRs for age 50–59 years: 7.27, 95% CI 2.33–22.55; age 60–69 years: 3.42, 95% CI 1.30–9.10). Three‐quarters of bladder cancers were diagnosed below the age of 70 years, representing an SIR of 5.03 (95% CI 2.62–9.67) vs the UK general population below the age of 70 years [7]. Pathological details were known for 10/12 respondents with bladder cancer —all these tumours were high‐grade non‐muscle‐invasive bladder cancer (NMIBC; five) or muscle‐invasive bladder cancer (MIBC; five).

This is the first UK study to quantify the association between exposure to certain groups of explosives and bladder cancer (BC). SIRs indicate an excess of BC occurrence, representing 27% of cancer diagnoses in ATs, a considerable excess compared to the UK general population [7]. Smoking is another contributor to BC risk, and BC cases from this study were either ex‐smokers (50%) or non‐smokers (50%) compared with other cancer cases (3% smokers, 61% ex‐smokers, 36% non‐smokers) and the no cancer group (2% smokers, 54% ex‐smokers, 44% non‐smokers). Hence, smoking does not appear to be the principal cause of the BC excess amongst this group. In addition, two younger diagnoses (age <40 years) may be linked to schistosomiasis (one veteran) or other causes (one, length of service <5 years). Male preponderance was expected given that females did not join the AT profession until 2001.

This small dataset does not permit robust multivariate analyses of risk factors and is indicative. Future studies should collect detailed information on a larger number of participants, as well as potential confounders, to provide more precise CIs. However, the difference in BC risk between AT respondents and the UK general population appears to be profound. If we were to assume that the remainder of the 2300 veteran/non‐veteran AT who have served in the squadron were within the no cancer group, our findings still demonstrate an excess of BC (SIRs for age 50–59 years: 2.17, 95% CI 1.17–4.05; age 60–69 years: 1.02, 95% CI 0.28–3.68). However, we are unable to account for attrition due to death in service, from cancer, or other diseases within that cohort.

When assessing the types of explosive exposures, weekly exposure to nitro‐explosives during disposal was 2.8‐times more frequent for those diagnosed with BC compared to those diagnosed with other cancers. This suggests that exposure to ammunition at end of useful service life, degraded/unsafe ammunition, or the products of detonation (gaseous, particulate, or within the soil medium of demolition areas [8]) is indicative of increased risk. These procedures align with activities subjectively characterised as higher risk by AT military heads of service, as many nitro‐explosive precursors and their metabolites are closely related to known bladder carcinogens (e.g., aromatic amines and polycyclic aromatic hydrocarbons). Notably, ATs also deal with homemade explosives, synthesised from a variety of harmful chemical precursors, as well as military or commercial explosives.

This survey presents the first quantitative data for a small but vital profession to the UK's national security and highlights an urgent need for further investigations into military exposure to explosives and the risk of BC. Regular exposure to nitro‐explosives is associated with high incidence rates, especially for those AT between the ages of 50 and 69 years, with a median length of 25 years from first exposure to BC diagnosis. Therefore, safe exposure levels should be established by duty holders to inform the protection and post‐exposure monitoring of all military personnel. Although the AT profession is inherently dangerous, the longer‐term risks for veterans worldwide should not be ignored. Since completion of this initial survey, two further AT veterans have been diagnosed with BC, taking the number of known cases amongst 688 individuals to 14.

## Disclosure of Interests

Gareth Collett is an unpaid charity trustee for the Felix Fund Bomb Disposal Charity and former head of the UK Bomb Disposal Profession. Richard T. Bryan receives research funding from Cancer Research UK (CRUK), Wellcome Trust (UK) and Janssen (European Union), is a paid consultant for Informed Genomics Limited (UK) and Cystotech (Denmark) and is an unpaid charity trustee for Action Bladder Cancer UK (UK). Mieke Van Hemelrijck receives funding from CRUK, the European Organisation for Research and Treatment of Cancer (EORTC, Belgium), Bayer (Germany), National Institute for Health Research (UK), Medical Research Council (UK), Guy's Cancer Charity (UK), Royal Marsden Cancer Charity (UK), and Movember Foundation (Australia). She is an unpaid member of the Board of the EORTC.
